# Gut Microbiota Dysbiosis and Increased NLRP3 Levels in Patients with Pregnancy-Induced Hypertension

**DOI:** 10.1007/s00284-023-03252-w

**Published:** 2023-04-06

**Authors:** Jingjing Wu, Dongmei Zhang, Meijing Zhao, Xiaowei Zheng

**Affiliations:** 1 Department of Obstetrics, The Second Affiliated Hospital of Fujian Medical University, Quanzhou, Fujian China; 2 Clinical Laboratory, The Second Affiliated Hospital of Fujian Medical University, Quanzhou, Fujian China

## Abstract

Pregnancy-induced hypertension (PIH) is one of the most common diseases, causing high maternal morbidity and mortality. However, the correlation of gut microbiota in PIH has not been reported. Our aim was to characterize the intestinal microbiota of patients with PIH compared with healthy people. We analyzed and compared the gut microbiota communities in the feces of 28 PIH patients with pregnancy(not pre-pregnancy) body mass index (including height and weight)-matched healthy controls using 16S rRNA gene sequencing and then investigate the relationships among gut microbiota, cytokines, and PIH. Compared with the healthy group, microbial α diversity was lower in the PIH group, but not statistically significant different. At the phylum level, Firmicutes, Bacteroidetes, Proteobacteria, and Actinobacteria exhibited obvious differences between the PIH and control groups. LEfSe analysis found 33 differentially abundant taxa between the two groups. The production of pro-inflammatory cytokines in PIH serum or placenta tissues was higher than that of the control group. In addition to alterations in gut microbiota composition, we also found that the Bac_Prevotellaceae, Pre_Prevotella bacteria were positively correlated with NLRP3 level, but negatively correlated with Bac_Bacteroidaceae, Bac_Bacteroides. PIH patients had gut microbiota dysbiosis and increased NLRP3 levels, which will lead to a better understanding of the relationship between the gut microbiota and PIH.

**Supplementary Information:**

The online version contains supplementary material available at 10.1007/s00284-023-03252-w.

## Introduction

Pregnancy-induced hypertension (PIH), defined as a systolic blood pressure > 140 mmHg and/or a diastolic blood pressure > 90 mmHg, refers to the new onset of hypertension with or without proteinuria, including hypertension, preeclampsia (PE), and eclampsia [1, 2]. Hypertension and complication are the most common medical problem in pregnancy, affecting up to 10% of pregnant women, causing high incidence rate and mortality of pregnant and parturient [3]. In the USA, 15% of maternal mortality is caused by PIH and its complications, which is the second leading cause of death in this population [4]. Although genetic, immune, metabolic susceptibility and other background investigations have been used in the study of PIH [5], the specific pathogenesis remains unclear.

Systemic inflammatory response may represent the pathological mechanism of PIH [5]. The IL-1β and IL-18, belonging to the member of the pro-inflammatory IL-1 cytokine superfamily, are enhanced in hypertension [6]. Inflammasomes belong to a large immune multiprotein complexes, which can strictly regulate the secretion of IL-1β and IL-18 [7]. Omi et al. suggested that the activated NLRP3 inflammasome could produce more chemokines, which was associated with a higher risk of hypertension [8]. PE patients usually show abnormalities in cholesterol, uric acid crystals, extracellular DNA, extracellular cell debris, advanced glycation end products, and free fatty acids. These abnormalities can activate NLRP3 inflammasome [9]. However, the mechanism of NLRP3 activation in pregnancy-induced hypertension has not been reported.

Gut microbiota is a complex and huge microbial community living in the digestive tract, and pregnancy is associated with changes in gut microbiota homeostasis [10, 11]. Omry et al. pointed out that the diversity of gut microbiota decreased significantly in the third trimester (T3) and the increase of Proteobacteria and Actinobacteria [12]. More and more evidence shows that the intestinal flora disorder in pregnant women is an important driving factor of hypertension and complications [13]. Liu et al. showed that there were differences in the gut microbiota between healthy and PE women, and in the PE patients, they suggest that there is a significant structural shift of the gut microbiota in PE patients [14]. The results were the same as in the rat model of preeclampsia superimposed on chronic hypertension. In healthy pregnant mice, a transient-specific dysregulation of gut ecology occurs in late pregnancy as the body adapts to pregnancy and allows full fetal growth, while this normal gut microbiological change does not occur in PIN rats [15]. Another study found that abnormal changes in gut ecology may be one reason for the increased inflammation and hypertension in the T3 [16]. Chen X et al. reported that dysbiosis influences the mother’s blood pressure and that gut *Fusobacterium* translocates into the placenta, causing local placental inflammation [16, 17], and butyrate-producing bacteria clearly cause blood pressure dysregulation and intestinal barrier dysfunction in the T3 of PE pregnancy [18]. A hyperactive NLRP3 inflammasome improves the gut symbiosis through gut microbiota increasing Tregs induction. Additionally, some microbial biomarkers of these diseases have been found, and the fecal microbiota targeting strategy has been recommended as a powerful tool for early diagnosis and treatment [19]. In recent years, studies have found that gut microbiota dysbiosis can contribute to intestinal barrier disorder and bacterial translocation and then cause persistent systemic inflammation and promote the occurrence and development of diseases [20, 21].

Studies have found that the imbalance between NLRP3 inflammasome and intestinal microecology as well as bacterial ectopia can mutually promote and aggravate the inflammatory response of the body, including NOD, NOD2, LRR, ASC, NLRP6 and IL-10 [22]. Intestinal microbial imbalance causes changes in gene expression in intestinal epithelial cells and activates the NLRP3 inflammasome [23], and leads to increased intestinal permeability and allows endotoxin (LPS) and other toxins to enter the body and cause inflammation [24]. In the present research, the difference of microbial population between PIN and non-PIN was detected by 16S rRNA, and the differential microbial flora was screened to explore the interaction with cytokines and NLRP3 inflammasome.

## Methods

This study was conducted at The Second Affiliated Hospital, Fujian Medical University, from January 2019 to December 2020 and was approved by the ethics review board of The Second Affiliated Hospital, Fujian Medical University (NO: 2020-221). Signed informed consents were obtained from all subjects on enrolment for the use of their data and samples for scientific purposes.

The inclusion criteria for PIH patients were women who matched the diagnostic criteria of the reference for PIH: A single blood pressure measurement was performed after a 5-min rest with the patient sitting at arm and heart level and classified enrolled blood pressure as blood pressure > 140 mmHg and/or a diastolic blood pressure > 90 mmHg [25]. The inclusion criteria were as follows: singleton pregnancy; All pregnant women had no antibiotics used, no history of chronic hypertension, diabetes, heart disease, chronic nephritis, systemic lupus erythematosus and other medical and surgical complications. The exclusion criteria were as follows: multiple pregnancy, fetal abnormalities, chronic hypertension, autoimmune diseases, gingivitis, and smoking. Fecal samples were collected at 31–41-week gestation, and the serum samples were collected at the same time.

Thirty cases were collected in both the experimental group and the control group, but when 16sRNA sequencing was carried out, the quality control of two samples in the experimental group failed to meet the standard, so only 28 formal samples were tested. A total of 58 singleton pregnancies were received in this study. Fecal samples were collected from 28 PIH patients, and 30 healthy controls matched with age, weight and blood pressure to analyze the intestinal flora and cytokine levels. The clinical information including age, mode of production, body weight, systolic blood pressure, diastolic blood pressure, albumin, urinary protein, gestational age and fetal weight, and total protein was recorded.

### Sample Collection, 16S rRNA Sequencing and Quantitative PCR Assay

Fecal samples were collected with feces collection containers, a catheter with screw cap, and each catheter was preloaded with 5 mL RNA stable solution. Samples were collected in tubes and then stored at – 80 °C until DNA extraction.

PCR amplification of the bacterial 16S rRNA genes V4 region was performed using the forward primer 515F (5′-GTGCCAGCMGCCGCGGTAA-3′) and the reverse primer 806R (5′-GGACTACHVGGGTWTCTAAT-3′). Sample-specific paired-end 6-bp barcodes were incorporated into the TrueSeq adaptors for multiplex sequencing [26]. The PCR components contained 25 μL of Phusion High-Fidelity PCR Master Mix, 3 μL (10 uM) primers, 10 μL DNA template, DMSO 3 μL, and ddH_2_O 6 μL. Thermal cycling consisted of initial denaturation at 98 °C for 30 s, followed by 25 cycles consisting of denaturation at 98 °C for 15 s, annealing at 58 °C for 15 s, and extension at 72 °C for 15 s, with a final extension of 1 min at 72 °C. PCR amplicons were purified with Agencourt AMPure XP Beads (Beckman Coulter, Indianapolis, IN) and quantified using the PicoGreen dsDNA Assay Kit (Invitrogen, USA). After the individual quantification step, amplicons were pooled in equal amounts, and pair-end 2 × 150 bp sequencing was performed using the Illumina NovaSeq6000 platform at GUHE Info technology Co., Ltd (Hangzhou, China). Additionally, extraction of general RNA from approximately 50 mg of placental tissue using TRIzol (Invitrogen, USA) following the corporation's directions. Briefly, using reverse transcription kit to synthesize cDNA, the primers directed at detecting mRNA expression were showed in Sup. Table 1. The normalization of relative gene expression levels was used GAPDH through the 2^−ΔΔCt^ comparative approach. And See supplemental documentation for primer validation.

Sequence data analyses were mainly performed using QIIME (v1.9.0, 10.1038/nmeth.f.303) and R packages (v3.2.0). Sequences with a distance-based similarity of 97% or greater were grouped into operational taxonomic units (OTUs, Vsearch v2.4.4) using the Usearch algorithm [27]. According to the sequence frequency, we detected the representative sequences for each OTU, which were aligned using the PyNAST algorithms. OTU-level ranked abundance curves were generated to compare the richness and evenness of OTUs among samples. The rarefaction curve is used to calculate the expected value of each alpha diversity index by extracting n Reads (n is less than the total number of measured Reads) based on the known relative content of each OTUs in the measured sequences. It can be used to compare the richness of species in samples with different amounts of sequencing data, and it can also be used to illustrate whether the amount of sequencing data of samples is reasonable. Beta diversity analysis was performed to investigate the structural variation of microbial communities across samples using UniFrac distance metrics and visualized via principal coordinate analysis (PCoA) and nonmetric multidimensional scaling (NMDS) [28–30].

LEfSe (10.1186/gb-2011-12-6-r60) analysis was applied to identify differentially abundant bacterial taxa between these two groups. Only those taxa that obtained a log linear discriminant analysis (LDA) score > 2 were ultimately considered.

### Cytokines Assay

Serum samples were collected, and the levels of cytokines were detected by ELISA assay according to the manufacturer’s protocol. The plasma was collected, centrifuged at 3000 rpm, 4 °C for 5 min, and then harvested the supeeen cytokines and bacteria, and then we rnate to determine the expressions of IL-1β, IL-6, TNF-α, IL-8 and IL-10 using ELISA kits following the corporation's instructions. Finally, the absorbance value was got in 450 nm using enzyme standard instrument.

### Statistical Analyses

In order to study the relationship between changes in intestinal microbial population and cytokines and inflammatory bodies NLRP3 in patients with PIN, clinical information and fecal samples were collected at 31–41 weeks of gestation, as well as serum samples and placentas. Stool was sequenced by 16SrRNA for microbial diversity analysis, and serum and placenta were tested for cytokines and inflammatory body NLRP3 levels. Clinical data and SPSS 19.0 were used for statistical analysis. Pearson analysis was used for correlation analysis. The statistics were expressed as the mean ± S.D. of three separate experiments. *p* value < 0.05 was considered statistically significant.

## Results

### The Basic Characteristics of PIH Group and Healthy Control Group

The baseline characteristics of the PIH group and the control group are summarized in Table 1. Compared with the control group, PIH group had significant differences in mode of production, BMI, systolic blood pressure, diastolic blood pressure, albumin, urinary protein, gestational age, fetal weight, and baby weight 10 percentile (*p* < 0.05). There was no difference in age and total protein (*p* > 0.05).

**Table 1:** The basic characteristics of PIH group and healthy control group

		PIH group (*n* = 30)	control group(*n* = 30)	*p*
Mode of production	Eutocia	8	28	< 0.001***
	Caesarean section	22	2	
Age		30.80 ± 4.11	29.07 ± 2.64	0.124
BMI		29.557 ± 4.341	26.287 ± 2.829	< 0.001***
Systolic blood pressure(mmHg)		163.13 ± 16.14	109.03 ± 7.54	< 0.001***
Diastolic blood pressure(mmHg)		105.17 ± 8.71	72.63 ± 3.92	< 0.001***
Total protein(g/L)		59.14 ± 6.67	60.47 ± 7.25	0.240
Albumin(g/L)		30.13 ± 3.63	33.51 ± 3.56	0.006**
Urinary protein(g/L)		1872.23 ± 2258.18	191.27 ± 60	< 0.001***
Gestational age (Weeks)		35.53 ± 2.21	39.03 ± 0.98	< 0.001***
Fetal weight(g)		2339.3 ± 507.8	3369.3 ± 234.0	< 0.001***
Baby weight percentiles(P10)		1542(1040,3600)	3000(2850,3850)	< 0.001***

Baby weight percentiles (a,b) means 10 percentile baby weight (the lowest weight, the highest weight)

** indicates significant difference between PIN group and non-PIN group

*** indicates very significant difference between PIN group and non-PIN group

### Gut Microbiota in the PIH Group and the Healthy Control Group

**Fig. 1:**
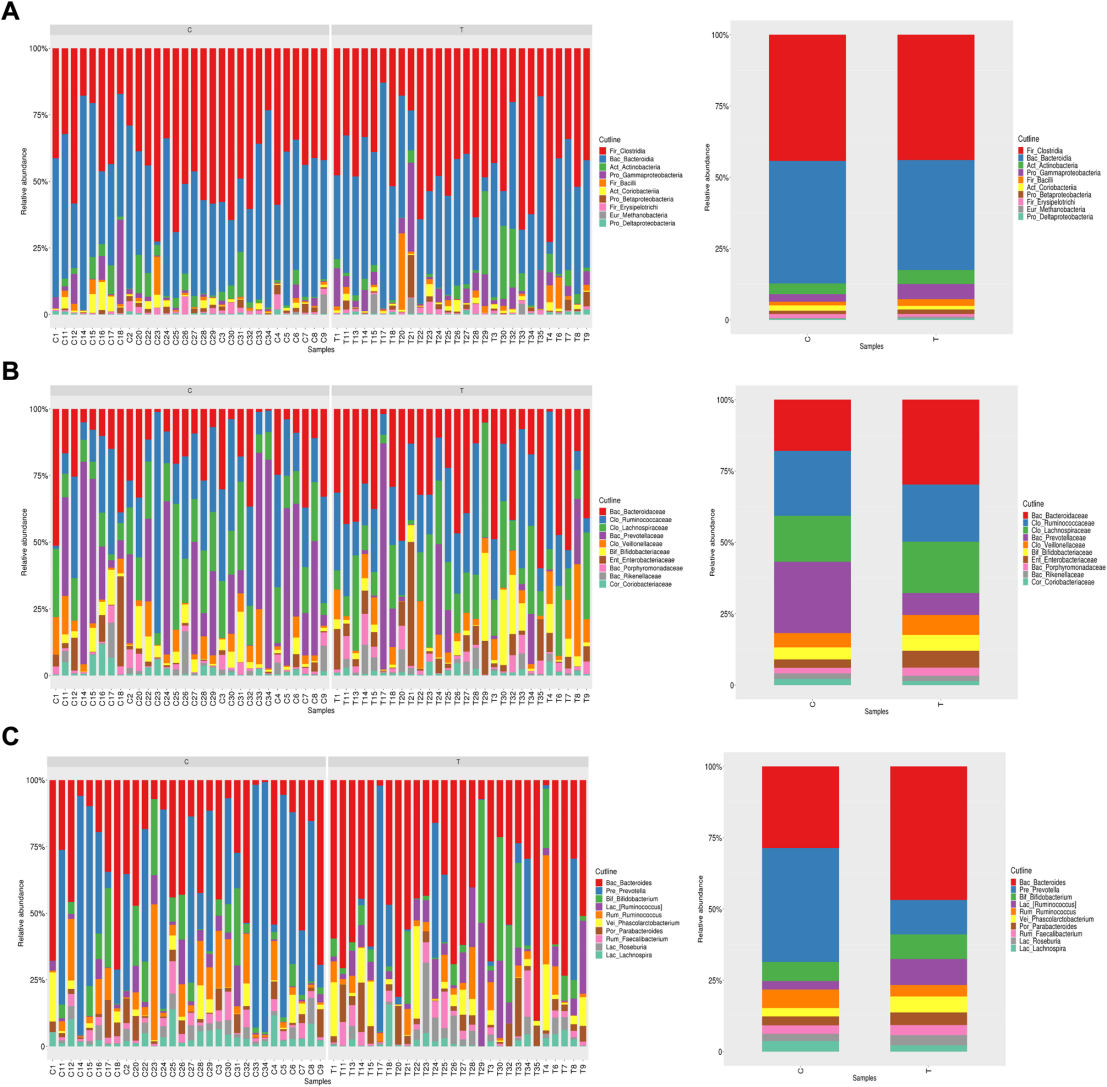
The relative abundance of gut microbiota was significantly different between the PIH group and the control group. **A** Class level; **B** Family level; **C** Genus level. Data represent the mean ± S.D of three independent experiments. C is Control group, T is PIH group

A total of 58 fecal samples were suitable for sequencing. Only the relative abundance of TM7_TM7-3 was significantly different between the two groups at the class level (*p* = 0.0037). Besides, there were eleven significantly different kinds of bacteria such as Bac_Bacteroidaceae at the family level (*p* < 0.05). There were fifteen significantly different bacteria, such as Bac_Bacteroides at the genus level (Fig. 1).

### α and β Diversity Between the PIH Group and the Control Group

**Fig. 2:**
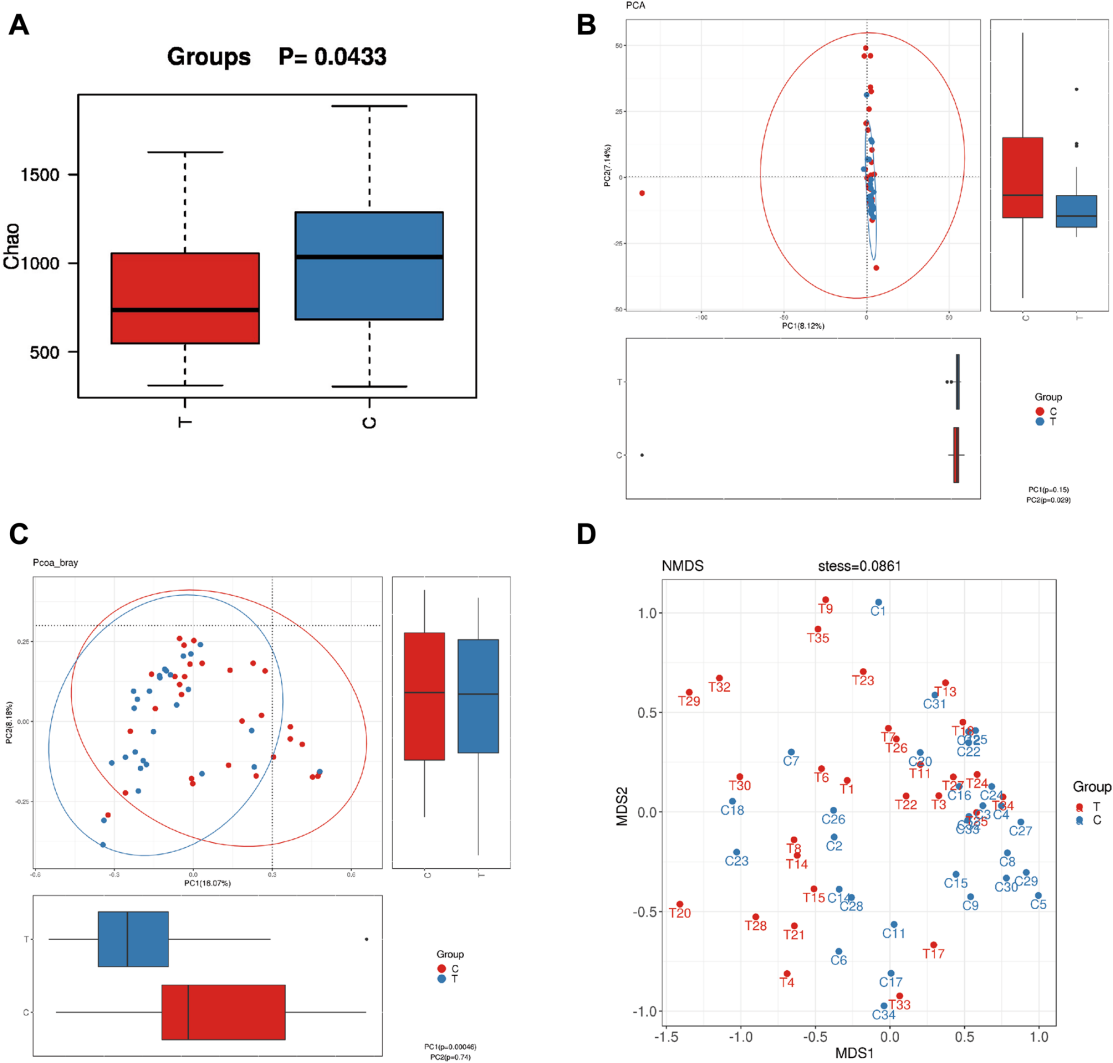
The significant differences in alpha and beta diversity analyses between the PIH pregnancy samples and the normal pregnancy group. **A** Chao index. **B** PCoA of Weighted UniFrac distance. **C** Bray–Curtis dissimilarity based PCoA plots. **D**. NMDS, non-metric multidimensional scaling. Chao index means one of α diversity. PCoA of Weighted UniFrac distance, Bray–Curtis dissimilarity and NMDS means beta diversity. Data represent the mean ± S.D of three independent experiments. C is Control group, T is PIH group. There is a significant difference between two groups of samples (*P* < 0.05)

The intestinal microbiota diversity of PIN group and healthy control group was estimated by Shannon diversity index method and Simpson diversity method, and the richness was estimated by phobicity method and Chao 1 method. The shape of the sparse curves indicates that new phylotypes are expected to be discovered by further sequencing. However, during the current sequencing process, the exponential curves of diversity reached a plateau for all samples, indicating that most of the diversity had been captured (sup. Fig. 1A, C, E). The Shannon diversity index (*p* = 0.1466, sup. Fig. 1B) showed that microbial α diversity is similar between groups. As shown in sup. Fig. 1D, there was no statistically significant difference in the results of Simpson diversity index (*p* = 0.6375). However, the Chao index (*p* = 0.0433) (Fig. 2A and sup. Fig. 1F) is statistically significant differences.

PCoA graph according to weighted UniFrac distance analysis was used for estimation β diversity. The red and blue dots represent the intestinal microbial samples of the control group and PIH group, respectively. Besides, a separation between these two groups could be observed from PC1 and PC2 scores, which account for 8.12% and 7.14% of the total variation, respectively (as shown in Fig. 2B, C). NMDS analysis indicated that there were significant differences in bacterial flora composition between these two groups (*p* < 0.05) (Fig. 2D).

### Taxonomic Biomarkers Between the PIH Group and the Healthy Control Group

**Fig. 3:**
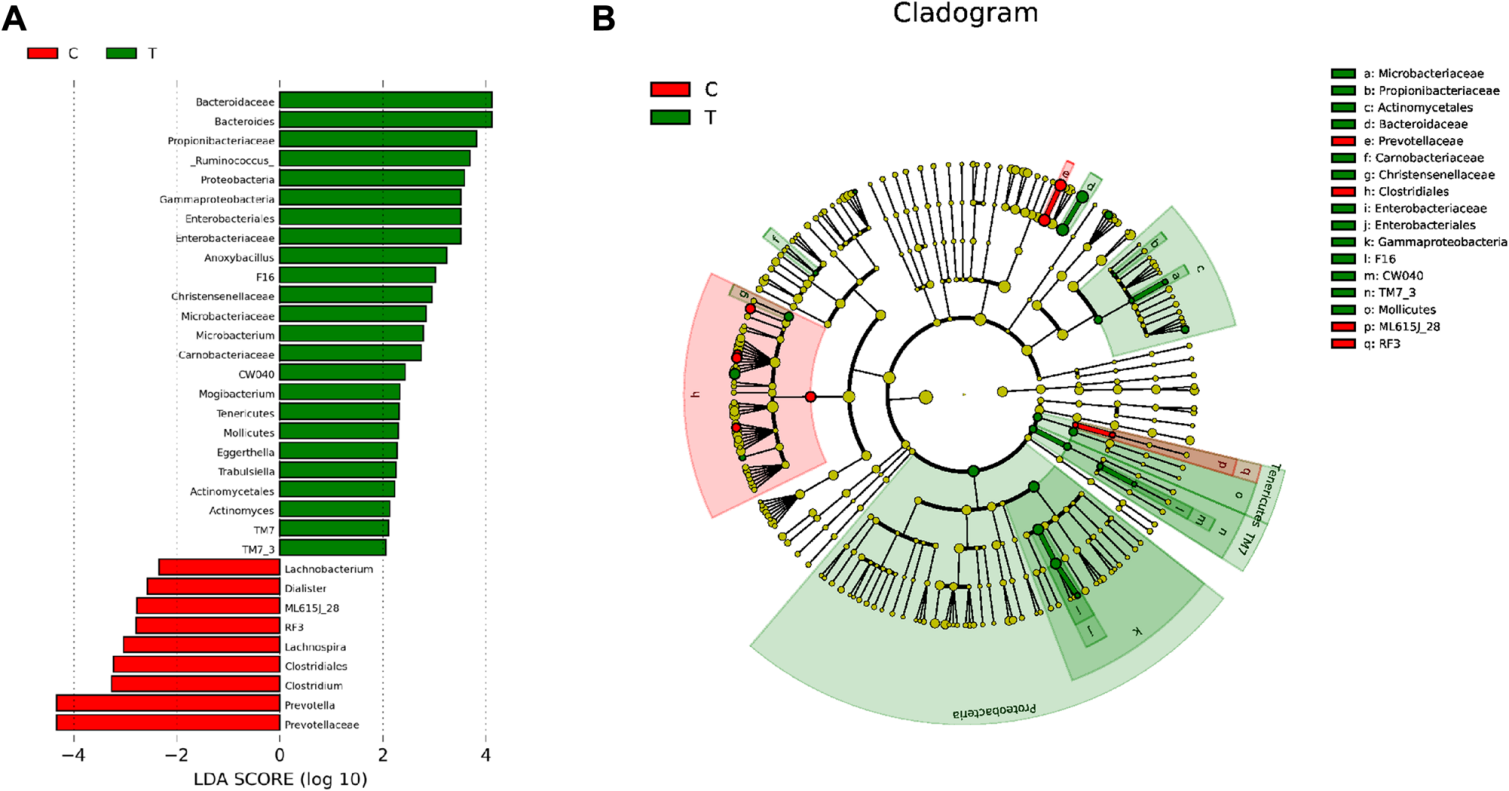
Bacterial taxa differences between the PIH group and the healthy control group using LEfSe analysis. **A** Scores and **B** Cladogram of taxonomic biomarkers identified by LDA using LEfSe in the PIH group and the healthy control group. The LDA scores (log10) > 2. Red and green colors represent a decrease and increase of abundance in the PIH group, respectively. C: the control group; T: the PIH group (Color figure online)

LEfSe analysis was applied to investigate the biomarkers among the two groups. We found 33 differentially abundant taxa between the two groups, all of which had a log LDA score > 2. From the Scores (Fig. 3A), the relative abundances of phylum of *TM7*, class of *TM7_3* and *Mollicutes*, order of *Actinomycetales,* genus of *Actinomyces*, *Trabulsiella* and *Eggerthella* were higher in the PIH group than those in the healthy control group, while the relative abundances of order of *Clostridiales*, family of Prevotellaceae and *Prevotella*, genus of *Clostridium*, etc., were lower in the PIH group than those in the healthy control group. Results are presented with red and green colors indicating a decrease and increase of abundance in the PIH group, respectively. The cladogram (Fig. 3B) showed that there were significant differences in abundance of class of *Gammaproteobacteria*, *TM7-3*, *Mollicutes*, *RF3*, order of *Actinomycetales*, *Enterobacteriales*, *Clostridiales*, *ML615J-28*, family of *Microbacteriaceae*, *Propionibacteriaceae*, *Bacteroidaceae*, *Carnobacteriaceae*, *Christensenellaceae*, *Enterobacteriaceae*, *F16*, *CW040*, *Prevotellaceae* between PIN group and healthy control group.

### Elevated Levels of Pro-Inflammatory Cytokines in Patients with PIH

To determine the correlation between the composition of the microbiome and the degree of inflammation, we measured the levels of proinflammatory cytokines in the serum of the PIN group and healthy controls.

**Fig. 4:**
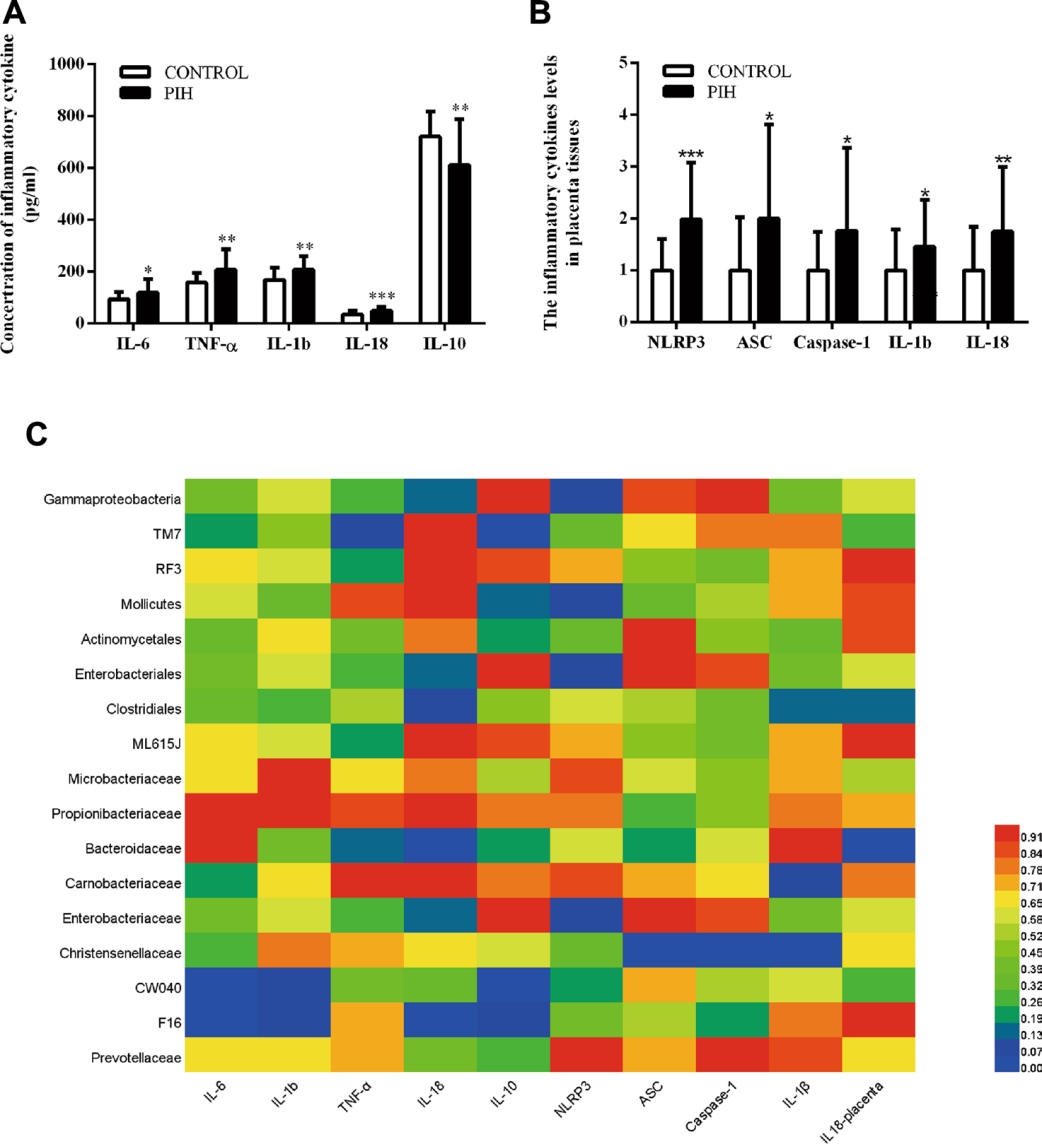
The cytokines levels in the serum and placenta tissues have significant difference between the PIH group and the control group, and the correlation coefficients between gut microbiota and cytokines in the two groups. **A** The inflammatory cytokines levels in serum. **B** The inflammatory cytokines levels in placenta tissues. **C** Correlation coefficients between gut microbiota and cytokines. The color means p-value in the (C). Data represent the mean ± S.D of three independent experiments. *P < 0.05 versus Control group

The pro-inflammatory cytokines secretion (IL-6, IL-1β, IL-18 and TNF-α) in PIH serum was higher than that of the control group, but the expression of IL-10 was decreased in PIH group (Fig. 4A). Similarly, the expression of inflammatory cytokines (NLRP3, ASC, Caspase-1, IL-1β, and IL-18) in placenta tissues was also increased in the PIH group compared with the healthy control group (Fig. 4B).

### Correlation Coefficients Between Gut Microbiota and Cytokines

In order to understand the relationship between intestinal microbiota and inflammation, we conducted correlation analysis (Fig. 4C, Table 2) between the marker gut microbiota in cladogram (Fig. 3B) and cytokines in serum and placenta (Fig. 4A). And then, we selected relevant intestinal gut microbiota and cytokines for correlation analysis (*p* < 0.05) (sup. Fig. 2). Between the PIH group and the control group (Fig. 4C), the results of correlation analysis between gut microbiota and cytokines suggested that *Bacteroidaceae* were positively correlated with IL-18 level in the serum and placenta tissues (sup. Figure 2A, B). And *Christensenellaceae* may have more influence on placenta, and it is positively correlated with ASC, Caspase-1 and IL-1βexpression, respectively (sup. Fig. 2C–E). *CW040* was positively correlated with IL-6 level but negatively correlated with IL-10 level in the serum (sup. Fig. 2F, G). And in the serum, IL-6 level also positively correlated with *F16* (sup. Fig. 2H). In the PIN group, we first deleted the gut microbiota with no correlation between cytokines and bacteria, and then we found that IL-1b level was positively correlated with *Prevotellaceae* in the serum, and TNF-α level was negatively correlated with *Gammaproteobacteria*, *Enterobacteriales,* and *Enterobacteriaceae* in the serum, respectively*.* In the placenta, expression of NLRP3 had a positive correlation with relative abundance of *Prevotellaceae* and a negative correlation with relative abundance of *Bacteroidaceae*, and *Christensenellaceae* relative abundance was positively correlated with ASC expression and IL-1β expression. There was a positive correlation between the expression of IL-18 in the placenta and *Bacteroidaceae* relative abundance.

**Table 2:** Correlation coefficients between gut microbiota and cytokines in the PIN group

	*Gammaproteobacteria*	*Enterobacteriales*	*Bacteroidaceae*	*Carnobacteriaceae*	*Christensenellaceae*	*Enterobacteriaceae*	*Prevotellaceae*
IL-6							
R	− 0.232	− 0.230	− 0.114	0.098	− 0.214	− 0.230	0.317
P	0.235	0.240	0.564	0.621	0.275	0.240	0.100
N	28	28	28	28	28	28	28
IL-1b							
R	− 0.331	− 0.329	− 0.075	0.108	− 0.143	− 0.329	0.465*
P	0.085	0.088	0.703	0.584	0.467	0.088	0.013
N	28	28	28	28	28	28	28
IL-10							
R	0.084	0.082	− 0.204	0.195	− 0.026	0.082	0.043
P	0.672	0.677	0.297	0.321	0.894	0.677	0.830
N	28	28	28	28	28	28	28
TNF-α							
R	− 0.385*	− 0.383*	0.009	− 0.063	− 0.105	− 0.383*	0.340
P	0.043	0.044	0.964	0.751	0.596	0.044	0.077
N	28	28	28	28	28	28	28
IL-18							
R	0.212	0.216	0.366	− 0.098	− 0.098	0.216	− 0.024
P	0.279	0.269	0.055	0.619	0.619	0.269	0.904
N	28	28	28	28	28	28	28
NLRP3							
R	0.206	0.212	− 0.440*	− 0.169	0.076	0.212	0.423*
P	0.293	0.280	0.019	0.389	0.699	0.280	0.025
N	28	28	28	28	28	28	28
ASC							
R	− 0.107	− 0.112	0.212	− 0.024	0.382*	− 0.112	− 0.151
P	0.588	0.572	0.278	0.904	0.045	0.572	0.445
N	28	28	28	28	28	28	28
Caspase-1							
R	− 0.169	− 0.179	− 0.012	− 0.015	0.404*	− 0.179	− 0.055
P	0.389	0.362	0.953	0.941	0.033	0.362	0.782
N	28	28	28	28	28	28	28
IL1β							
R	− 0.232	− 0.240	− 0.100	0.298	0.564**	− 0.240	0.039
P	0.234	0.219	0.613	0.123	0.002	0.219	0.845
N	28	28	28	28	28	28	28
IL18-placenta							
R	− 0.339	− 0.335	0.408*	− 0.093	− 0.111	− 0.335	− 0.140
P	0.078	0.081	0.031	0.639	0.573	0.081	0.478
N	28	28	28	28	28	28	28

* indicates a correlation between cytokines and gut microbes

## Discussion

PIH was one of the most common disease during pregnancy and formed one deadly triad along with hemorrhage and infection, causing high maternal morbidity and mortality [31]. It is reported that the incidence rate of PIH in India is between 5 and 15% [32]. While many theories have been developed about the pathogenesis of PIH, including placental implantation abnormalities, vascularization or metabolic factors [33, 34], its mechanism has not been well elucidated.

Gut microbiota has many functions and plays key roles in systemic immunity and metabolism [35]. The change of gut microbiota is closely related to the occurrence of many diseases, which may be the target of therapeutic intervention [35]. However, the relationship between the intestinal flora disturbance and PIH was rarely studied. Here we report a significant imbalance of intestinal flora in PIH patients [36].

In our results, alpha diversity indices of the fecal microbiota were similar between PIN group and no PIN group, which was similar to other’s studies [11, 37]. It has been reported that intestinal microbial diversity decreased in infancy, subsequently developing eczema [38, 39]. Moreover, the beta diversity of PIH patients was significantly different from that of the control group; it indicated a significant difference in microbial diversity between the PIN and healthy controls. In the study, the abundances of Bacteroidetes, Proteobacteria were increased, while the abundance of Firmicutes was decreased in PIH group. Bacteroidetes are the largest phylum of Gram-negative bacteria inhabiting our gastrointestinal tract and are considered the leading players of the healthy state and sophisticated homeostasis safeguarded by gut microbiota. It plays vital roles in immune disorders, and systemic diseases including metabolic syndrome and also neurological disorders [40, 41]. And then, we used LEfSe to screen microbial markers. We found that the relative abundance of *Propionibacteriaceae*, *Bacteroidaceae*, *Carnobacteriaceae*, *Christensenellaceae*, *Enterobacteriaceae*, *F16* and *CW040* increased at the family level, while the relative abundance of Prevotellaceae decreased. We found that maternal and infant symptoms in the PIN group were associated with changes in microbial composition and abundance at the family level. For example, Propionibacteriaceae [42], Bacteroidaceae [43], and Prevotellaceae [44] can produce short-chain fatty acids under the action of propionibacteriaceae in vivo, and short-chain fatty acids such as propionic acid and butyric acid participate in glucose metabolism. Prevotellaceae [45] and Christensenellaceae [46] are an obesity-related microbe that affects host BMI through metabolism. TM7 (F16 and CW040 belong to the order TM7) is common in oral flora and can be used as a predictor of secondary cardiovascular events [47]. Women with preeclampsia are more likely to develop cardiovascular disease later in life [48], and intestinal microbial disturbances predict PIN patients are more likely to develop disease during pregnancy and can be used as a candidate microbial marker.

Dysregulation of microbial composition can lead to inflammation, while low-grade intestinal inflammation can disrupt the intestinal microbiota [49]. PIN is associated with chronic inflammation, mitochondrial dysfunction and fetal death [50]. In our result, the proinflammatory cytokines (IL-6, IL-1β, IL-18 and TNF-α) increased in the circulation and the placenta tissue, while anti-inflammatory cytokines IL-10 decreased. Previous studies revealed that proinflammatory cytokines might be potential predictors in the prognosis of PIH [51]. Interestingly, we found that NLRP3 expression was elevated in the PIH group, and the main mechanism is that the activation of the inflammatory body NLRP3 plays an important role in the maturation of inflammatory cytokines. It is considered to be an important part of the human natural immune system and is closely related to the occurrence of type II diabetes, hypertension, female genital tract inflammation and other inflammatory diseases [52]. NLRP3 can be activated by a variety of exogenous and endogenous stimulatory signals, and autooligomerization occurs, which can collect adaptin ASC and caspase-1 to form mature inflammasome. The latter lyse inactive proinflammatory cytokine precursors pro-IL-1β and pro-IL-18 into mature IL-1β and IL-18, promoting the maturation and secretion of IL-1β and IL-18, leading to inflammation. It has been found that the imbalance of inflammasome and intestinal microecology as well as bacterial ectopic can promote each other and aggravate the inflammatory response of the body [53]. Its mechanism is related to the imbalance of intestinal flora, the increase in the rate of opportunistic pathogens and the decrease in the symbiotic ratio, especially the decrease in the number of bacteria. The protective intestinal barrier affects the gene expression of intestinal epithelial cells, leading to the increase of intestinal permeability and the introduction of endotoxin into the body. After the identification of immune cells, a variety of inflammatory factors will be produced [54]. Recent literature suggests that intestinal microecological imbalance may be involved in the activation of NLRP3 inflammasome. Its mechanism is related to the intestinal flora regulating the activation of multiple pattern recognition receptors, thereby causing changes in a series of signaling pathways [55]. Therefore, we investigated whether the changes in gut microbes in PIN patients are related to the activation of NLRP3 inflammasome. Correlation analysis found that the expression of Prevotellaceae was positively correlated with NLRP3 and negatively correlated with Bacteroides. Bacteroides are a double-edged sword. As members of the Polysaccharide Degradation Consortium, they help release energy from dietary fibers and starches. However, they are also involved in the release of toxic products during the protein breakdown process. Bacteroides are enriched in women with type 1 diabetes at 3 months of pregnancy and are part of the LPS bacterial population and may be involved in regulating the NLRP3 inflammasome to control inflammation. Studies have shown that butyrate produced by Prevotellaceae can regulate pro-inflammatory cytokines IL-1β and TNF-α through NF-κB signaling [56], Prevotellaceae was also closely related to inflammation in mice with colitis [57], Ffar2 signaling in colon cancer regulates intestinal health by regulating the abundance of Prevotellaceae, and SCFA receptors Ffar2 and Hcar2 induce inflammatory body activation to balance colon Treg cell homeostasis [58]. Based on correlation analysis, it was speculated that Prevotellaceae could activate NLRP3 inflammasome to regulate inflammation. Intestinal bacteria can activate NLRP3 inflammasome, which activates a large number of inflammatory cytokines through the blood circulation and enhances the level of central inflammatory response [35, 36].

In conclusion, we believe that Prevotellaceae and Bacteroidaceae can be used as candidate microbial markers of PIN to regulate inflammation and promote the progression of PIN by activating NLRP3 inflammasomes.

## Conclusion

We speculate that the alterations of gut microbiota composition may contribute to NLRP3 activation and then accelerate the inflammatory response in PIH patients.

## Supplementary Information

Below is the link to the electronic supplementary material.

Supplementary file1 (DOCX 295 KB)

Supplementary file2 (DOCX 861 KB)

## Data Availability

The datasets used and/or analyzed during the current study available from the corresponding author on reasonable request. Submission ID: SUB10992010; BioProject ID: PRJNA800478. The project information will be accessible with the following link: http://www.ncbi.nlm.nih.gov/bioproject/800478.

## Abbreviations

PIH: Pregnancy-induced hypertension
IL-1β: Interleukin-1β
IL-18: Interleukin-18
