# Supplementary material for: Gut Microbiota Dysbiosis and Increased NLRP3 Levels in Patients with Pregnancy-Induced Hypertension
Source: Curr Microbiol. 2023 Apr 6;80(5):168. doi: 10.1007/s00284-023-03252-w (PMC10079714; doi:10.1007/s00284-023-03252-w)
Supplement: Supplementary file 1 — Supplementary file1 (DOCX 295 KB) [file 284_2023_3252_MOESM1_ESM.docx]

**Experimental design:**

1. Definition of experimental and control groups

Experimental group: The inclusion criteria for PIH patients were women who matched the diagnostic criteria of the reference for PIH: A single blood pressure measurement was performed after a 5-minute rest with the patient sitting at arm and heart level and classified enrolled blood pressure as blood pressure > 140 mmHg and/or a diastolic blood pressure > 90 mmHg. Control group: non-PIN pregnancy women.

1. Number within each group

28 PIH patients and 30 healthy controls. In pin group, 2 samples were found to be unqualified when two samples were missing for 16S sequencing.

**Sample:**

1. Description

placenta tissue

1. Microdissection or macrodissection

Macrodissection

1. Processing procedure

Placental tissues from pregnant women who gave birth were included in the experiment after individual consent.

1. If frozen, how and how quickly?

Placentas were placed in cryopreserved tubes, 1g each, followed immediately by liquid nitrogen.

1. If fixed, with what and how quickly?

Not Applicable

1. Sample storage conditions and duration (especially for FFPEb samples)

After flash freezing in liquid nitrogen, it was stored in the -80℃ refrigerator for half a year.

**Nucleic acid extraction：**

1. Procedure and/or instrumentation

Automatic snowflake ice machine，vortex mixer，Frozen high speed centrifuge

1. Name of kit and details of any modifications

Tissue RNA extraction by NucleoZol method

1. Details of DNase or RNase treatment

Using the RNase-free centrifuge tube， RNase-free water

**Contamination assessment (DNA or RNA)**

1. Instrument and method

Nano microspectrophotometer

1. Nucleic acid quantification and Purity (A260/A280)

| Control group | concentration(ng/µl) | 260/280 | 260/230 |
| --- | --- | --- | --- |
| 1 | 1987 | 1.94 | 2.27 |
| 2 | 820 | 2.14 | 1.79 |
| 3 | 180 | 1.99 | 1.88 |
| 4 | 300 | 1.91 | 2.01 |
| 5 | 1673 | 1.70 | 1.52 |
| 6 | 430 | 1.82 | 1.40 |
| 7 | 917 | 2.07 | 2.08 |
| 8 | 937 | 1.90 | 1.97 |
| 9 | 1095 | 2.18 | 1.61 |
| 10 | 650 | 2.29 | 2.05 |
| 11 | 1312 | 2.13 | 2.09 |
| 12 | 920 | 2.16 | 1.92 |
| 13 | 1220 | 2.09 | 2.12 |
| 14 | 744 | 2.23 | 2.17 |
| 15 | 1717 | 2.09 | 2.32 |
| 16 | 1471 | 1.90 | 1.90 |
| 17 | 1700 | 2.08 | 2.18 |
| 18 | 500 | 2.28 | 1.56 |
| 19 | 1700 | 2.01 | 1.96 |
| 20 | 1100 | 2.03 | 1.74 |
| 21 | 1800 | 2.06 | 2.09 |
| 22 | 935 | 1.96 | 1.48 |
| 23 | 1430 | 2.00 | 1.78 |
| 24 | 1165 | 1.94 | 1.98 |
| 25 | 875 | 1.94 | 1.58 |
| 26 | 955 | 1.95 | 1.90 |
| 27 | 924 | 1.97 | 1.92 |
| 28 | 711 | 1.94 | 1.51 |
| 29 | 920 | 1.98 | 1.38 |
| 30 | 1400 | 1.94 | 1.53 |
| PIN group | concentration(ng/µl) | 260/280 | 260/230 |
| 1 | 650 | 1.93 | 1.58 |
| 2 | 400 | 1.90 | 1.85 |
| 3 | 2000 | 1.95 | 1.76 |
| 4 | 1500 | 1.96 | 2.12 |
| 5 | 855 | 1.96 | 2.00 |
| 6 | 1150 | 1.90 | 2.02 |
| 7 | 1300 | 1.91 | 1.63 |
| 8 | 1240 | 1.97 | 2.08 |
| 9 | 550 | 1.90 | 1.81 |
| 10 | 1250 | 1.97 | 2.14 |
| 11 | 1210 | 1.98 | 1.59 |
| 12 | 1120 | 1.99 | 1.97 |
| 13 | 2000 | 1.94 | 2.00 |
| 14 | 2000 | 1.94 | 1.83 |
| 15 | 1200 | 1.90 | 1.84 |
| 16 | 1200 | 1.95 | 2.00 |
| 17 | 1460 | 1.95 | 1.53 |
| 18 | 1230 | 1.94 | 1.91 |
| 19 | 1500 | 1.92 | 2.02 |
| 20 | 2000 | 1.84 | 1.75 |
| 21 | 450 | 1.94 | 1.52 |
| 22 | 1110 | 1.93 | 1.31 |
| 23 | 1400 | 1.97 | 2.00 |
| 24 | 1110 | 1.96 | 1.99 |
| 25 | 1200 | 1.96 | 1.92 |
| 26 | 1300 | 1.95 | 2.09 |
| 27 | 1300 | 1.93 | 1.81 |
| 28 | 1700 | 1.98 | 2.04 |

**Reverse transcription:**

Reverse transcription system (20 μl) :

Phase I: Total RNA was denatured and unstranded

RNA X μl（0.5~1 μg）

oligo（dT） 0.5 μl

RNase free ddH_2_O Y μl

5 μl

The reagent was added as above, and the mixture was mixed by microcentrifugation for 10 s. The mixture was bathed in water for 5 min at 70 ° C and then immediately bathed in ice for 5 min.

Phase 2: Reverse transcription reaction

5×Reaction Buffer 4 μl

MgCl_2_ 3 μl

PCR Nucleotide Mix 1 μl

Reverse Transcriptase 1 μl

RNAase inhibitor 0.5 μl

RNase free ddH_2_O 5.5 μl

Total 15 μL

RNAase inhibitor reagent was added as described above, and the mixture was mixed by microcentrifugation for 10 s, followed by 25 ℃ for 5 min and 42℃ for 60 min. 70 ℃ for 15 min.

**qPCR target information**

1. Gene symbo：NLRP3、ASC、Caspase-1、IL-1β、IL-18
2. Sequence accession number：

NLRP3 Gene ID: 114548

ASC Gene ID: 29108

Caspase-1 Gene ID: 834

IL-1β Gene ID: 3553

IL-18 Gene ID: 3606

1. Primer information--Location of amplicon and Amplicon length

**NLRP3**

**Primer pair**

|  | **Sequence (5'->3')** | **Template strand** | **Length** | **Start** | **Stop** | **Tm** | **GC%** | **Self complementarity** | **Self 3' complementarity** |
| --- | --- | --- | --- | --- | --- | --- | --- | --- | --- |
| **Forward primer** | GCTGGCATCTGGGGAAACCT | Plus | 20 | 10 | 29 | 62.51 | 60.00 | 3.00 | 0.00 |
| **Reverse primer** | CTTAGGCTTCGGTCCACACA | Minus | 20 | 101 | 82 | 59.68 | 55.00 | 3.00 | 0.00 |
| **Product length** | 92 | | | | | | | | |

**ASC**

**Primer pair**

|  | **Sequence (5'->3')** | **Template strand** | **Length** | **Start** | **Stop** | **Tm** | **GC%** | **Self complementarity** | **Self 3' complementarity** |
| --- | --- | --- | --- | --- | --- | --- | --- | --- | --- |
| **Forward primer** | GATCCAGGCCCCTCCTCA | Plus | 18 | 367 | 384 | 60.04 | 66.67 | 4.00 | 1.00 |
| **Reverse primer** | AAGAGCTTCCGCATCTTGCT | Minus | 20 | 558 | 539 | 60.04 | 50.00 | 4.00 | 1.00 |
| **Product length** | 192 | | | | | | | | |

**Caspase-1**

**Primer pair**

|  | **Sequence (5'->3')** | **Template strand** | **Length** | **Start** | **Stop** | **Tm** | **GC%** | **Self complementarity** | **Self 3' complementarity** |
| --- | --- | --- | --- | --- | --- | --- | --- | --- | --- |
| **Forward primer** | GCCTGTTCCTGTGATGTGGA | Plus | 20 | 1049 | 1068 | 59.96 | 55.00 | 3.00 | 0.00 |
| **Reverse primer** | TTCACTTCCTGCCCACAGAC | Minus | 20 | 1223 | 1204 | 59.89 | 55.00 | 3.00 | 1.00 |
| **Product length** | 175 | | | | | | | | |

**IL-1β**

**Primer pair**

|  | **Sequence (5'->3')** | **Template strand** | **Length** | **Start** | **Stop** | **Tm** | **GC%** | **Self complementarity** | **Self 3' complementarity** |
| --- | --- | --- | --- | --- | --- | --- | --- | --- | --- |
| **Forward primer** | CAGAAGTACCTGAGCTCGCC | Plus | 20 | 92 | 111 | 60.18 | 60.00 | 6.00 | 1.00 |
| **Reverse primer** | AGATTCGTAGCTGGATGCCG | Minus | 20 | 244 | 225 | 59.97 | 55.00 | 4.00 | 2.00 |
| **Product length** | 153 | | | | | | | | |

**IL-18**

**Primer pair**

|  | **Sequence (5'->3')** | **Template strand** | **Length** | **Start** | **Stop** | **Tm** | **GC%** | **Self complementarity** | **Self 3' complementarity** |
| --- | --- | --- | --- | --- | --- | --- | --- | --- | --- |
| **Forward primer** | TGACCAAGGAAATCGGCCTC | Plus | 20 | 359 | 378 | 60.04 | 55.00 | 4.00 | 0.00 |
| **Reverse primer** | ATGGTCCGGGGTGCATTATC | Minus | 20 | 430 | 411 | 59.89 | 55.00 | 4.00 | 1.00 |
| **Product length** | 72 | | | | | | | | |

**qPCR protocol**

1. Complete reaction conditions

| Stage1 | Reps: 1 | 95℃ | 5 | min |
| --- | --- | --- | --- | --- |
| Stage2 | Reps: 40 | 95℃ | 10 | sec |
|  |  | 60℃ | 30 | sec |
|  |  | 72℃ | 30 | Sec |
| Stage3 | Reps: 1 | 95℃ | 15 | sec |
|  |  | 60℃ | 60 | sec |
|  |  | 95℃ | 15 | sec |

1. real-time PCR reaction system

| 反应原料 | reaction system |
| --- | --- |
| 2x SYBR qPCR Master Mix | 12.5 µL |
| Primer 1 (10 µM) | 1.0 µL |
| Primer 2 (10 µM) | 1.0µL |
| cDNA | 2.0µL |
| ddH_2_O | Up to 25 µL |

Note：2x SYBR qPCR Master kit is namedpromegaA6001-GoTaq qPCR Master Mix and producted in PROMEGA. Manufacturer of qPCR instrument：CG-02/05 made in HEAL FORCE.

**qPCR validation**

| primer | standard curve | amplification efficiency | K value | R^2^ | Curve of amplification | solubility curve |
| --- | --- | --- | --- | --- | --- | --- |
| NLRP3 | 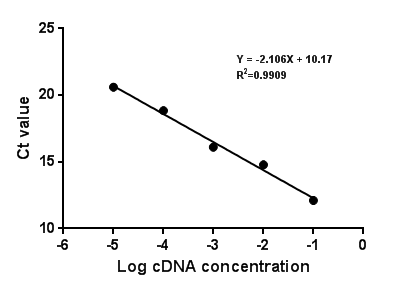 | 1.984240419 | -2.106 | 0.9909 | 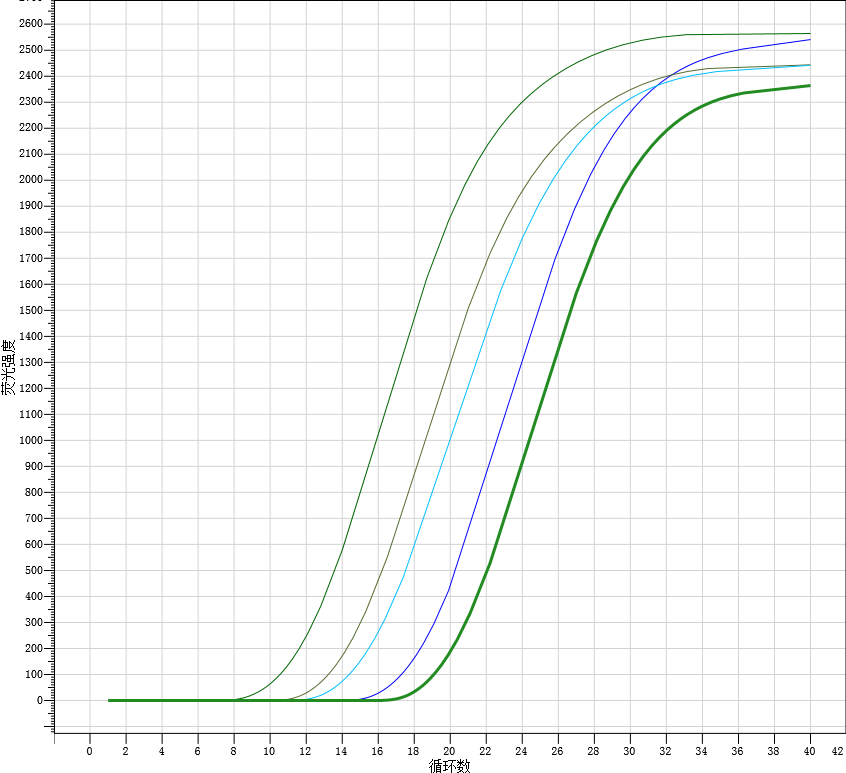 | 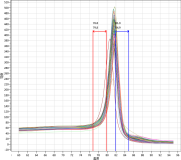 |
| ASC | 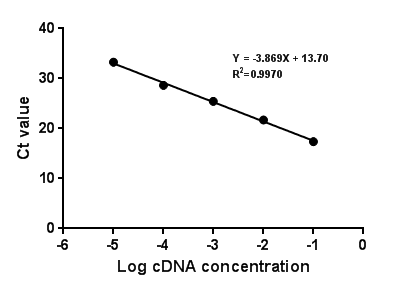 | 0.813279366 | -3.869 | 0.9970 | 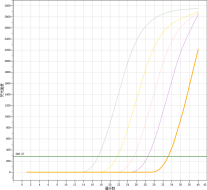 | 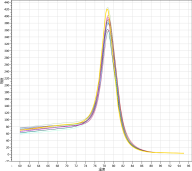 |
| Caspase-1 | 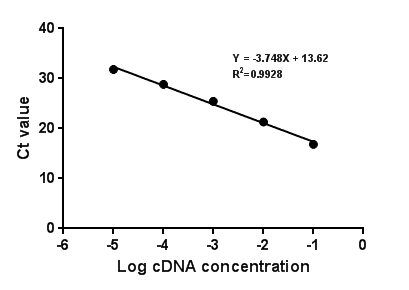 | 0.848455351 | -3.748 | 0.9928 | 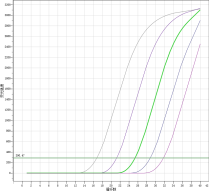 | 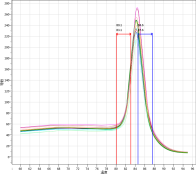 |
| IL-1β | 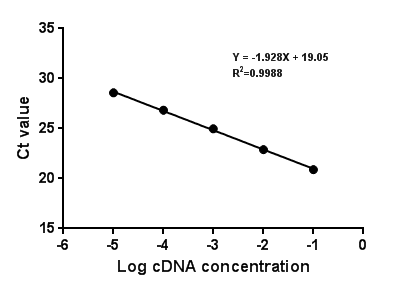 | 2.301202757 | -1.928 | 0.9988 | 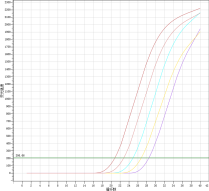 | 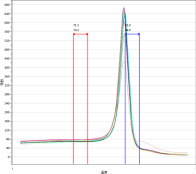 |
| IL-18 | 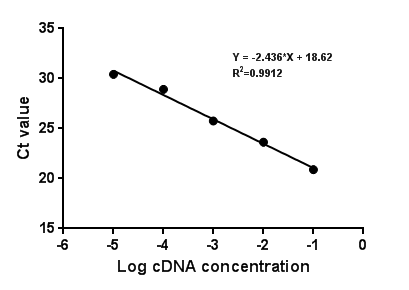 | 1.546882634 | -2.436 | 0.9912 | 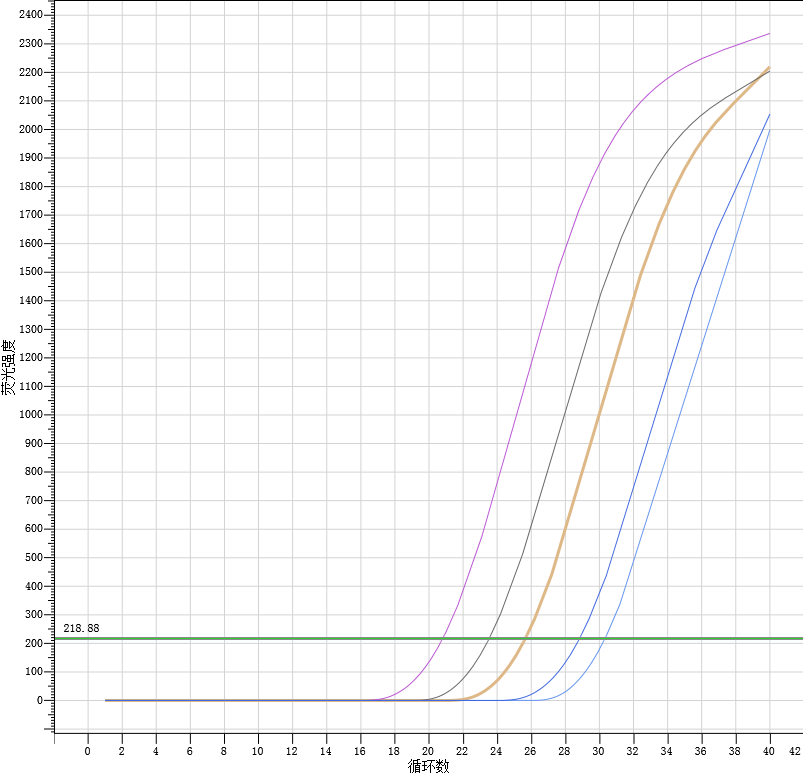 | 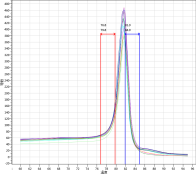 |

**Data analysis**

The relative quantitative analysis of the data was performed according to the 2^-ΔΔCt^ method, which was calculated as follows (x represents any sample) : ΔΔCt = (CT._target_-CT. _GAPDH_)X - (Ct._target_-Ct._GAPDH_)_Control_.
