# Supplementary material for: Gut Microbiota Dysbiosis and Increased NLRP3 Levels in Patients with Pregnancy-Induced Hypertension
Source: Curr Microbiol. 2023 Apr 6;80(5):168. doi: 10.1007/s00284-023-03252-w (PMC10079714; doi:10.1007/s00284-023-03252-w)

**Supplementary Material**

**Sup.Table 1 The primer sequences**

| NLRP3 | FORWARD | 5’- GCTGGCATCTGGGGAAACCT-3’ |
| --- | --- | --- |
|  | REVERSE | 5’- CTTAGGCTTCGGTCCACACA-3’ |
| ASC | FORWARD | 5’- GATCCAGGCCCCTCCTCA-3’ |
|  | REVERSE | 5’- AAGAGCTTCCGCATCTTGCT-3’ |
| Caspase-1 | FORWARD | 5’- GCCTGTTCCTGTGATGTGGA-3’ |
|  | REVERSE | 5’- TTCACTTCCTGCCCACAGAC-3’ |
| IL-1β | FORWARD | 5’- CAGAAGTACCTGAGCTCGCC-3’ |
|  | REVERSE | 5’- AGATTCGTAGCTGGATGCCG-3’ |
| IL-18 | FORWARD | 5’- TGACCAAGGAAATCGGCCTC-3’ |
|  | REVERSE | 5’- ATGGTCCGGGGTGCATTATC-3’ |
| GAPDH | FORWARD | 5’- AGAAGGCTGGGGCTCATTTG-3’ |
|  | REVERSE | 5’- AGGGGCCATCCACAGTCTTC-3’ |

**Sup. Fig. 1. α diversity between the PIH group and control group. (A).** Shannon index Dilution curve. **(B).** Shannon index between these two groups. **(C).** Simpson index Dilution curve. **(D)**. Simpson index between these two groups. **(D).** Chao index Dilution curve. **(F).** Chao index between these two groups. The intestinal microbiota diversity of PIN group and healthy control group was estimated by Shannon diversity index method and Simpson diversity method, and the richness was estimated by phobicity method and Chao 1 method. And the exponential curves of diversity reached a plateau for all samples, indicating that most of the diversity had been captured. Data represents the mean ± S.D of three independent experiments. C is Control group, T is PIH group.There is a significant difference between two groups of samples (*P* < 0.05).


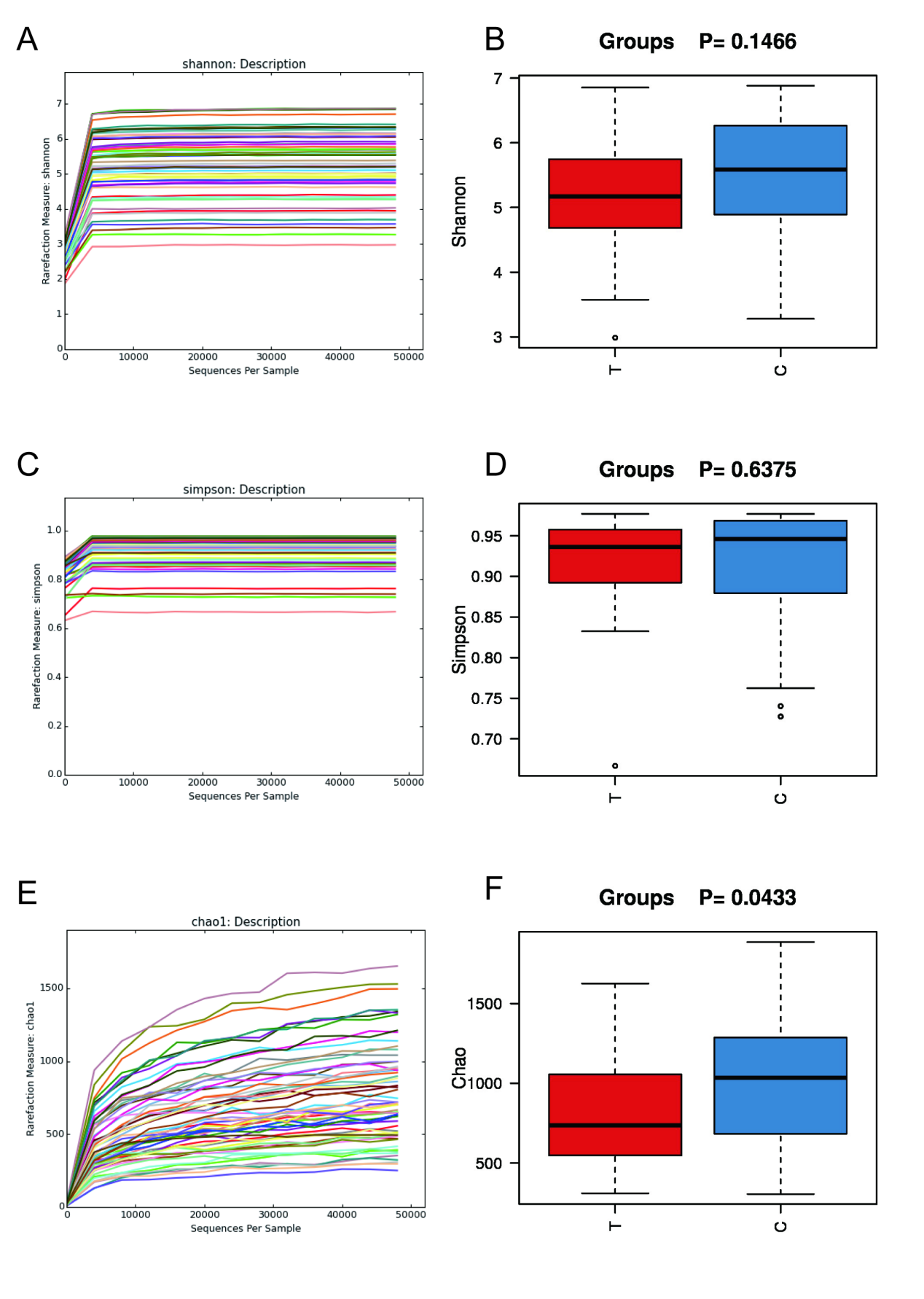


**Sup. Fig. 2. Correlation coefficients between gut microbiota and cytokines in the two groups.** Correlation analysis of gut bacteria and cytokines. r>0.2 means there is a correlation, and * meas P<0.05, ** meas P<0.01.


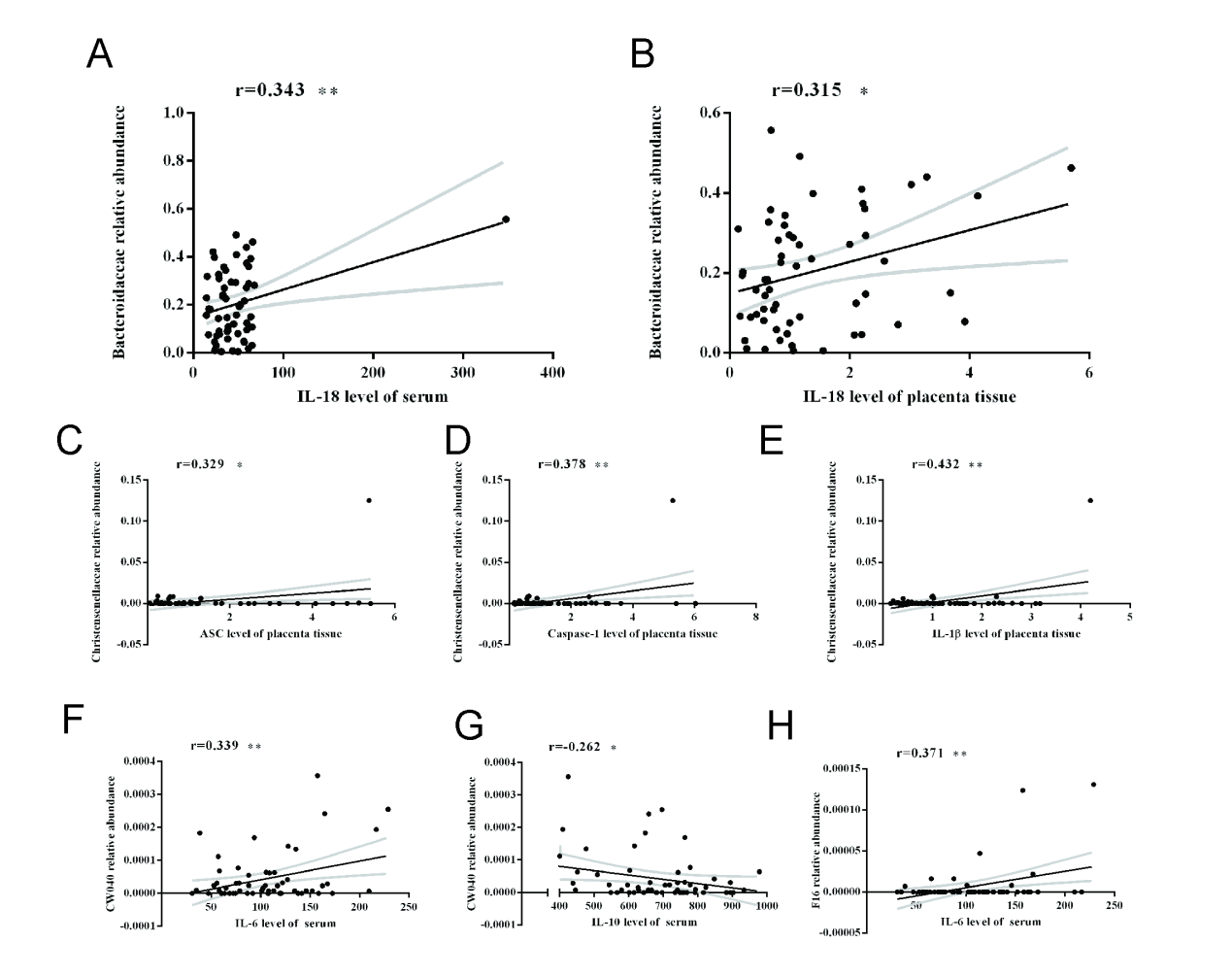

Supplement: Supplementary file 2 — Supplementary file2 (DOCX 861 KB) [file 284_2023_3252_MOESM2_ESM.docx]
